# Circulating expression and clinical significance of LncRNA ANRIL in diabetic kidney disease

**DOI:** 10.1007/s11033-022-07843-x

**Published:** 2022-09-21

**Authors:** Yanting Zhu, Lixia Dai, Xiangyou Yu, Xintian Chen, Zhenjiang Li, Yan Sun, Yan Liang, Bing Wu, Qiong Wang, Xiaoming Wang

**Affiliations:** 1 Center of Nephropathy and Hemodialysis, Shaanxi Provincial People’s Hospital, 710068 Xi’an, Shaanxi P.R. China; 2 Department of Endocrinology, Shaanxi Provincial People’s Hospital, 710068 Xi’an, Shaanxi People’s Republic of China

**Keywords:** Diabetic kidney disease, Long-chain non-coding RNA, ANRIL, Gene expression

## Abstract

**Background:**

Long noncoding RNA ANRIL has been found to be involved in the pathogenesis of diabetic kidney disease (DKD) and is expected to be a new target for prevention of DKD. However, the circulating expression and clinical significance of ANRIL in DKD patients is uncertain. This study aims to explore this issue.

**Methods:**

The study consisted of 20 healthy controls, 22 T2DM patients (normalbuminuria) and 66 DKD patients (grouped as follows: microalbuminuria, n = 23; macroalbuminuria, n = 22 and renal dysfunction, n = 21). The expressions of ANRIL in peripheral whole blood of all participants were measured by RT-qPCR.

**Results:**

The expression of ANRIL was significantly up-regulated in DKD patients (microalbuminuria, macroalbuminuria and renal dysfunction groups) than that in healthy control group. ANRIL was also over-expressed in macroalbuminuria and renal dysfunction groups in comparison with normalbuminuria group. ANRIL expression was positively correlated with Scr, BUN, CysC, urine β2-MG and urine α1-MG; while negatively correlated with eGFR in DKD patients. In addition, ANRIL was the risk factor for DKD with OR value of 1.681. The AUC of ANRIL in identifying DKD was 0.922, and the sensitivity and specificity of DKD diagnosis were 83.3% and 90.5%, respectively.

**Conclusion:**

Our results indicated that highly expressed ANRIL in peripheral blood is associated with progression of DKD. Circulating ANRIL is an independent risk factor of DKD and has a highly predictive value in identifying DKD.

## Introduction

Diabetic kidney disease (DKD) is a serious microvascular complication of diabetes mellitus (DM) and a primary etiology for end-stage renal disease (ESRD) worldwide [1–3]. The pathophysiology of DKD is multifactorial in which several common mechanisms involved, such as glomerular and tubulointerstitial inflammation, dysregulated cellular apoptosis and changes in the extracellular matrix (ECM), leading to diabetic glomerular lesions, proteinuria, decreased glomerular filtration rate (GFR) and renal fibrosis [4]. Renal fibrosis is an important pathological feature of DKD, manifested as glomerulosclerosis, tubular interstitial fibrosis and vascular sclerosis, which is majorly caused by epithelial/endothelial-mesenchymal-transition (EMT/EndMT), resulting in abnormal activation of renal myofibroblasts and excessive accumulation of renal ECM [5]. The conventional therapeutics of DKD including RAS blockade and antidiabetic drugs (such as sodium-glucose co-transporter-2 inhibitors, glucagon-like peptide-1 receptor agonists, dipeptidyl peptidase-4 inhibitors) are only able to slow down the rate of progression but not stop or reverse the disease. New drugs targeting DN pathomechanisms have become a major focus for the development of new therapies, such as N-acetyl-seryl-aspartyl-lysyl-proline (AcSDKP), endothelin receptor antagonists, vitamin D receptor activators, pentoxifylline and antioxidants [6, 7].

Currently decreased GFR and albuminuria have long been considered significant manifestations of DKD [8, 9]. However, the effect of GFR and albuminuria on the early diagnosis of DKD is limited, since they cannot identify the DM patients who are at risk of microvascular complications before renal damage actually occurs. Therefore, it is important to explore a new biomarker for the early detection of DKD among DM to increase the time to manage the disease and improve clinical outcome.

Long-chain non-coding RNAs (lncRNAs), a novel class of nonprotein-coding functional RNA molecules with a transcript length greater than 200 nucleotides, are recognized as gene expression regulators and involved in a variety of physiological and pathological processes [10, 11]. Increasing evidence suggests that lncRNAs contribute to the pathogenesis of various diseases, including cancers, cardiovascular diseases and kidney diseases [12–15]. Studies have indicated that lncRNAs induce EMT/EndMT and renal fibrosis in DKD [16, 17]. In addition, lncRNAs adopt a secondary structure, which is relatively stable in body fluids, such as blood and urine. Therefore, lncRNAs could potentially serve as biomarkers for predicting the diagnosis and prognosis of diseases or targets for drug treatment. Gene therapy targeting lncRNAs, which is promising for modulating diseases at the genetic level and able to overcome the limitations of incompatible proteins, aims to treat disease by artificially controlling gene expression and is considered to be the “third generation” of therapeutic drugs [15]. However, it is still in the preclinical stage.

Antisense RNA to INK4 locus (ANRIL), also known as CDKN2B-AS1, is transcribed from the short arm of human chromosome 9 on P21, which is involved in cell proliferation, migration, apoptosis, inflammation, immune responses and DNA damage [18]. ANRIL has been found to play an significant role in the pathogenesis and development of cardiovascular diseases, type 2 diabetes, atherosclerosis and cancers [19–22]. ANRIL has been also shown to be a molecular marker in the diagnosis of ischemic stroke and an indicator to predict the development of diabetic retinopathy [23, 24]. Study has indicated that ANRIL knock-down suppresses mouse mesangial cell proliferation, fibrosis, inflammation via regulating Wnt/β-catenin and MEK/ERK pathways in DKD [25]. In addition, ANRIL silencing alleviates high glucose-induced inflammation, oxidative stress and apoptosis via upregulation of MME in podocytes [26]. However, the clinical significance of ANRIL in DKD is still unclear. This study aims to examine the expression of ANRIL in peripheral whole blood of DKD patients and to further explore the relationship between ANRIL and DKD, which provided a new theoretical basis for identifying new markers of lncRNAs in DKD patients.

## Materials and methods

### Participants

Patients diagnosed with type 2 diabetes mellitus (T2DM) based on the criteria of the American Diabetes Association (ADA) were collected from Shaanxi Provincial People’s Hospital (Xi’an, Shaanxi, China) between September 2020 and December 2021. Patients with type 1 diabetes, secondary diabetes, urinary tract infection, urolithiasis, pregnancy, superimposed systemic diseases and other glomerular diseases were excluded. 88 diabetic patients were enrolled in this study in which 22 were T2DM patients (patients with normalbuminuria, urine albumin creatinine ratio (UACR) < 30 mg/g) and 66 were DKD patients. DKD patients were divided into three groups according to UACR and serum creatinine: (1) Patients with microalbuminuria (UACR 30–300 mg/g), n = 23; (2) Patients with macroalbuminuria (UACR > 300 mg/g), n = 22; and (3) Patients with increased serum creatinine (renal dysfunction) (serum creatinine > 120µmol/L), n = 21. Meanwhile, 20 non-diabetic healthy volunteers were enrolled as control group. The present study was approved by the ethical committee for human investigation of Shaanxi Provincial People’s Hospital and was conducted according to the Declaration of Helsinki. Informed consent was obtained from all participants.

### Clinical data collection

General data were collected as follow: gender, age, body height, body weight, systolic blood pressure (SBP), diastolic blood pressure (DBP) and duration of diabetes on admission. Body mass index (BMI) was calculated using the formula: BMI = body weight/body height^2^ (kg/m^2^). The following laboratory parameters were obtained from each patient: glycated hemoglobin A1c (HbA1c), fasting serum glucose (FSG), UACR, urine β2-microglobulin (β2-MG), urine α1-microglobulin (α1-MG), serum creatinine (Scr), blood urea nitrogen (BUN), Cystatin C (CysC), neutrophil gelatinase-associated lipocalin(NGAL), uric acid (UA), white blood cell (WBC), hemoglobin (HGB), albumin (ALB), triglyceride (TG), total cholesterol (TC), high density lipoprotein (HDL) and low density lipoprotein (LDL) at the time of enrollment. UACR was calculated using the formula: UACR = urine albumin/creatinine. The eGFR was calculated according to the modified MDRD formula: eGFR = 186 × Scr^− 1.154^ × Age^− 0.203^ × gender (1 if male, 0.742 if female).

### Peripheral blood samples collection

Peripheral whole blood was collected by venipuncture with an ethylenediaminetetraacetic acid (EDTA) anticoagulant vacutainer from all patients and stored at − 80 °C until analysis. Total RNA was extracted as soon as possible.

### Quantitative real-time polymerase chain reaction

1mL peripheral whole blood was centrifuged at 3000rpm for 5min and the supernatants were removed. Then 3mL red cell lysis buffer added and mixed before being centrifuged at 3000rpm for 5min. The supernatant was then discarded and the extracted leukocytes were collected. The total RNA of leukocytes was extracted using Trizol reagent (Servicebio, Wuhan, China), and then dissolved in RNase-free water. The concentration of RNA was determined using NanoDrop 2000 (Thermo scientific, Waltham, MA, USA). Extracted RNA was reversibly transcribed into complementary DNA (cDNA) using Servicebio®RT First Strand cDNA Synthesis Kit (Servicebio, Wuhan, China). Quantitative real-time polymerase chain reaction was performed using SYBR Green qPCR Master Mix (Servicebio, Wuhan, China) on a CFX RT-PCR system (Bio-Rad). PCR reaction system was as follows: pre-degenerated at 95°C for 10min, followed by 40 cycles of 95°C for 15s and 60°C for 30s. The expression level of lncRNA ANRIL was normalized to the expression level of GAPDH as a housekeeping gene. The relative quantitative value was expressed by the 2^−ΔΔCt^ method. ANRIL primer sequences were shown as follows: upstream: 5’-AGGGTTCAAGCATCACTGTTAGG-3’; downstream: 5’-GAAACCCCGTCTCTACTGTTACCT-3’.

### Statistical analysis

SPSS software, version 18.0 (SPSS, Inc., Chicago, IL, USA) was used for statistical analysis. Normally distributed data were presented as mean ± S.D. and non-normally distributed data were expressed as median range. Differences between two groups for quantitative data and qualitative data were compared using a t test and chi-square test, respectively. Comparisons of normally distributed data in 3 or more groups were analyzed using one-way ANOVA, while non-normally distributed data were analyzed using nonparametric counterpart Kruskal-Wallis test. Correlations were examined using Pearson’s correlation analysis. Binary regression analysis was used to determine the influence factors of the presence of DKD. The diagnostic value of ANRIL was evaluated by ROC curve analysis. Area under the ROC curve (AUC) was calculated. When AUC = 0.5, diagnostic value was denied. The cut-off value and corresponding sensitivity and specificity were determined according to ROC curve analysis. P < 0.05 was considered to represent significant differences between groups.

## Results

### Clinical and biochemical characteristics of study populations

The basic characteristics of individuals involved in this study and results of biochemical analyses within the study groups were displayed in Table 1. Age, sex and BMI were matched for each group. There was no statistical differences in TG, TC, HDL and LDL among the all groups (P > 0.05). SBP and DBP were extremely higher in renal dysfunction group than in other groups (P < 0.01). Scr, BUN, CysC and NGAL levels in renal dysfunction group were also higher than those in other diabetic patients and healthy controls (P < 0.05). HGB and ALB were significantly decreased in renal dysfunction group compared to other diabetic patients and healthy controls (P < 0.05). EGFR was also significantly lower in renal dysfunction patients than other groups (P < 0.01). Slight significance was also observed in parameters such as UA and WBC between renal dysfunction patients and other four groups (P < 0.05). In addition, the disease duration was increased in DKD patients (microalbuminuria, macroalbuminuria and renal dysfunction groups) in comparison with DM patients (P < 0.05). UACR, urine β2-MG and urine α1-MG values of the macroalbuminuria and renal dysfunction groups were obviously higher than those of the normalbuminuria group (P < 0.01). Increased HbA1c was evident in renal dysfunction groups in comparison with normalbuminuria and microalbuminuria groups (P < 0.05). FSG were significantly higher in DM and all DKD patient than that in healthy control group (P < 0.01).

**Table 1:** The basic characteristics and clinical parameters of the study subjects. Data are presented as mean ± SD or number. BMI, body mass index; SBP, systolic blood pressure; DBP, diastolic blood pressure; HbA1c, glycated hemoglobin A1c; FSG, fasting serum glucose; UACR, urine albumin creatinine ratio; β2-MG, β2-microglobulin; α1-MG, α1-microglobulin; Scr, serum creatinine; BUN, blood urea nitrogen; CysC, Cystatin C; NGAL, neutrophil gelatinase-associated lipocalin; UA, uric acid; WBC, white blood cell; HGB, hemoglobin; ALB, albumin; TG, triglyceride; TC, total cholesterol; HDL, high density lipoprotein; LDL, low density lipoprotein; eGFR, estimated glomerular filtration rate. P < 0.05 indicated statistical significance

Parameter	Healthy control (n = 20)	Normalbuminuria (n = 22)	Microalbuminuria (n = 23)	Macroalbuminuria (n = 22)	Renal dysfunction (n = 21)	P
Age (years)	51.75 ± 12.20	52.27 ± 8.04	56.48 ± 11.17	55.68 ± 7.69	56.62 ± 8.49	0.287
Sex (Male/Female)	12/8	13/9	12/11	14/8	14/7	0.894
BMI (kg/m^2^)	24.39 ± 1.52	25.17 ± 4.01	25.48 ± 3.95	23.33 ± 2.84	23.58 ± 3.14	0.121
Disease duration (years)	NA	7.09 ± 4.67	11.70 ± 8.04	11.30 ± 6.25	15.81 ± 7.51	0.001^*^
SBP (mmHg)	125.90 ± 8.03	121.77 ± 10.76	136.30 ± 16.81	133.86 ± 19.12	150.76 ± 13.21	0.000^*^
DBP (mmHg)	71.75 ± 5.78	75.95 ± 7.92	79.52 ± 11.02	74.91 ± 9.6	85.19 ± 9.35	0.000^*^
HbA1c (%)	NA	8.04 ± 1.67	8.20 ± 1.81	8.84 ± 2.57	10.06 ± 3.47	0.037^*^
FSG (mmol/L)	4.82 ± 0.50	9.34 ± 3.31	9.30 ± 2.91	9.25 ± 3.25	9.51 ± 3.53	0.000^*^
UACR (mg/g)	NA	5.98 ± 5.32	93.13 ± 70.71	1642.74 ± 2660.59	3855.22 ± 2552.92	0.000^*^
Urine β2-MG (µg/mL)	NA	279.87 ± 524.43	555.31 ± 690.52	1020.30 ± 947.42	2058.60 ± 762.64	0.000^*^
Urine α1-MG (ng/mL)	NA	15.59 ± 16.81	32.10 ± 26.55	60.56 ± 30.84	94.71 ± 25.40	0.000^*^
Scr (µmol/L)	62.50 ± 12.68	54.95 ± 11.12	64.15 ± 21.87	71.69 ± 26.66	309.87 ± 198.37	0.000^*^
BUN (mmol/L)	5.00 ± 1.43	5.35 ± 1.17	5.45 ± 1.87	6.39 ± 1.63	15.67 ± 4.50	0.000^*^
CysC (mg/L)	0.90 ± 0.13	0.91 ± 0.20	1.20 ± 0.46	1.21 ± 0.33	3.13 ± 0.78	0.000^*^
NGAL (ng/mL)	120.60 ± 35.40	127.43 ± 52.49	152.83 ± 56.06	139.79 ± 65.47	375.85 ± 274.77	0.000^*^
UA (µmol/L)	329.71 ± 78.88	332.88 ± 91.48	350.95 ± 91.98	350.63 ± 96.29	417.12 ± 138.16	0.045^*^
WBC (10^9^/L)	5.62 ± 1.20	6.23 ± 1.33	6.50 ± 2.19	6.43 ± 1.37	7.55 ± 2.96	0.036^*^
HGB (g/L)	146.70 ± 13.24	145.59 ± 12.76	138.57 ± 16.86	128.55 ± 12.67	99.38 ± 20.18	0.000^*^
ALB (g/L)	43.34 ± 3.01	40.64 ± 2.95	39.52 ± 3.26	31.86 ± 7.64	28.48 ± 6.53	0.000^*^
TG (mmol/L)	1.76 ± 1.19	1.48 ± 0.73	2.34 ± 1.93	1.69 ± 0.88	2.09 ± 1.74	0.270
TC (mmol/L)	4.70 ± 0.92	4.03 ± 0.95	4.41 ± 1.43	5.11 ± 1.17	4.69 ± 1.43	0.068
HDL (mmol/L)	1.18 ± 0.37	0.99 ± 0.35	1.08 ± 0.26	1.18 ± 0.39	1.14 ± 0.41	0.425
LDL (mmol/L)	2.91 ± 0.76	2.47 ± 0.72	2.54 ± 0.88	2.96 ± 0.88	2.45 ± 0.89	0.130
eGFR (ml/min/1.73m^2^)	106.79 ± 12.73	109.70 ± 9.17	97.13 ± 22.77	91.61 ± 24.51	23.28 ± 11.82	0.000^*^

### Expression of circulating ANRIL in all groups

**Fig. 1:**
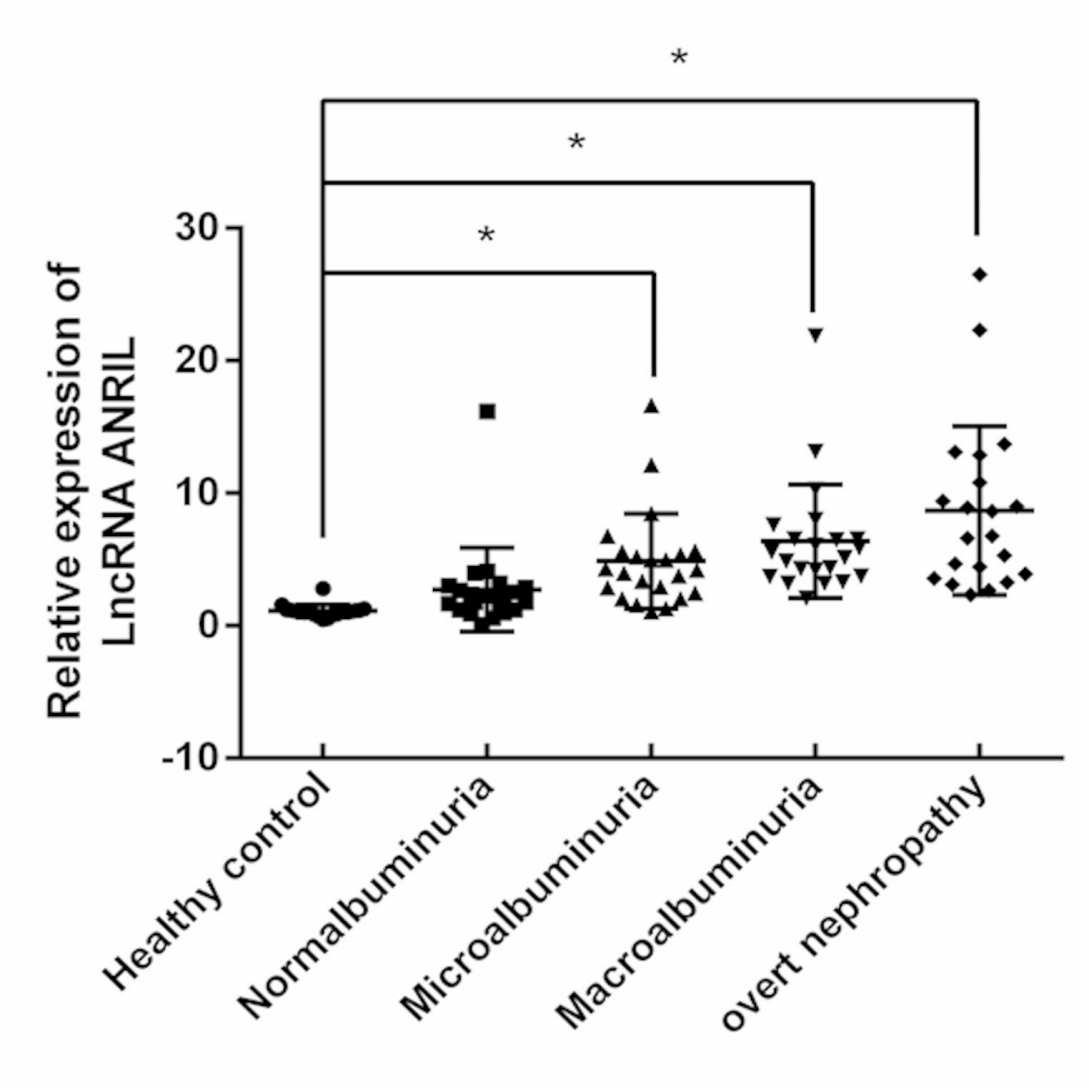
Expression of circulating ANRIL in all groups. The expression of ANRIL in peripheral blood was significantly upregulated in DKD patients than that in healthy control group. *P < 0.05 versus healthy controls

The differential expression of ANRIL in normalbuminuria, microalbuminuria, macroalbuminuria, renal dysfunction groups and healthy control group are shown in Fig. 1. The result indicates that the expression of ANRIL was significantly up-regulated in DKD patients (microalbuminuria, macroalbuminuria and renal dysfunction groups) than that in healthy control group (P < 0.01). In addition, ANRIL was over-expressed in macroalbuminuria and renal dysfunction groups in comparison with normalbuminuria group (P < 0.01). Whereas, there was no significant difference in ANRIL expression between normalbuminuria group and healthy controls (P = 0.199).

### Correlation between ANRIL expression and clinical parameters in DKD patients

**Fig. 2:**
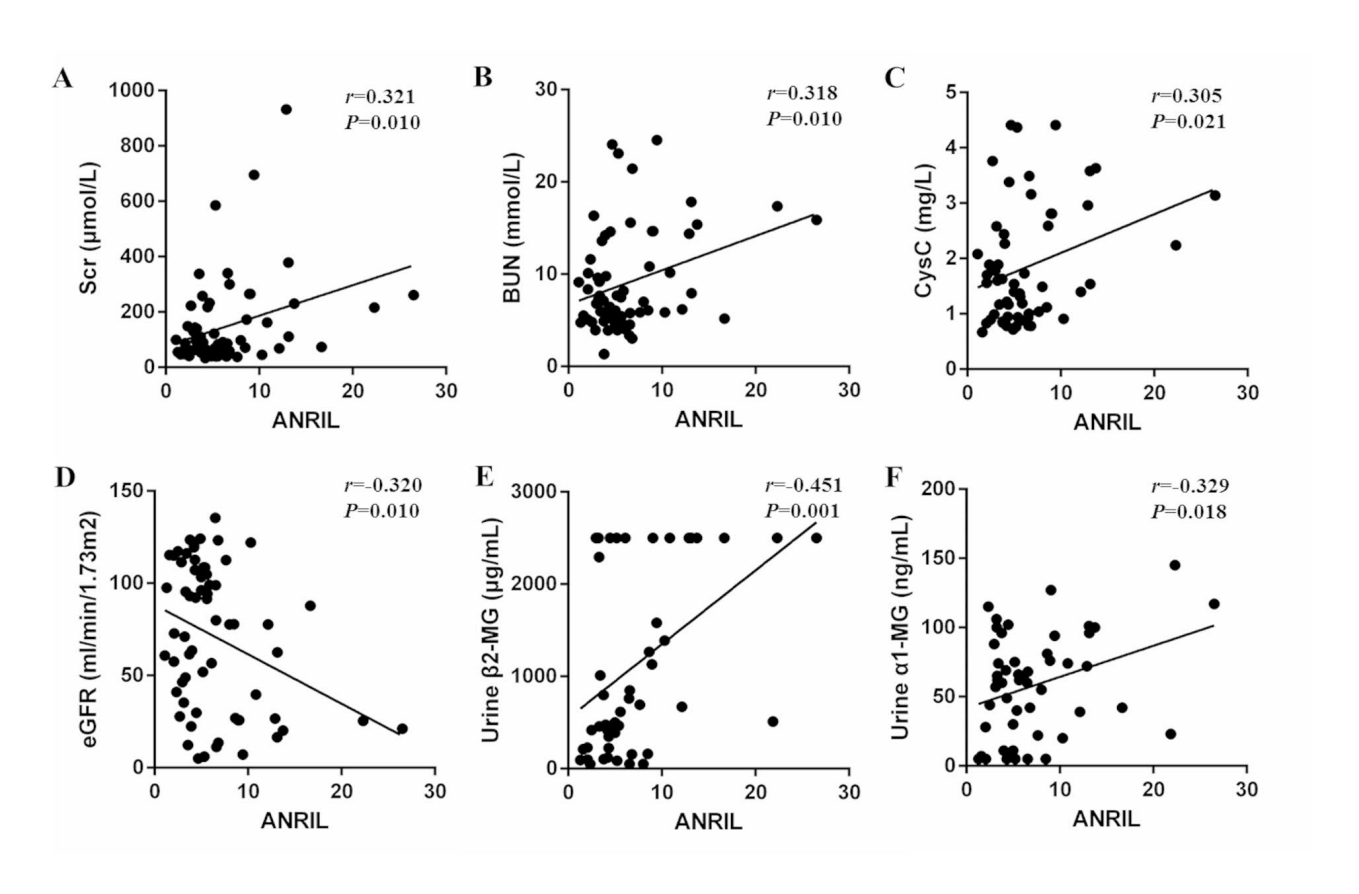
Correlation between ANRIL expression and Scr, BUN, CysC, urine β2-MG, urine α1-MG and eGFR in DKD patients

The correlations between ANRIL expression and clinical parameters in DKD patients were analyzed using Pearson’s linear correlation. Fig. 2 reveals that ANRIL expression was positively correlated with Scr, BUN, CysC, urine β2-MG and urine α1-MG in DKD patients (all P < 0.05). In addition, a negative correlation between ANRIL expression and eGFR was observed (P = 0.01) (Fig. 2). There were no correlation between ANRIL expression and BMI, disease duration, SBP, DBP, HbA1c, FSG, UACR, NGAL, UA, WBC, HGB, ALB, TG, TC, HDL, LDL.

### Influence factor in the presence of DKD

Binary regression analysis showed that ANRIL, SBP, α1-MG and disease duration were the risk factors of DKD, with OR value of 1.681, 1.248, 1.142 and 1.599 (P < 0.05); while, HGB was found to be the protective factor of DKD, with OR value of 0.838 (P < 0.05) (Table 2).

**Table 2:** Influence factor in the presence of DKD. SBP, systolic blood pressure; α1-MG, α1-microglobulin; HGB, hemoglobin; eGFR, estimated glomerular filtration rate. P < 0.05 was considered significant

Parameter	B	SE	Walds	P	OR	95%CI
ANRIL	0.519	0.219	5.633	0.018	1.681	1.095–2.582
SBP	0.222	0.079	7.948	0.005	1.248	1.070–1.457
α1-MG	0.133	0.049	7.361	0.007	1.142	1.038–1.258
HGB	-0.177	0.073	5.890	0.015	0.838	0.726–0.966
Disease duration	0.470	0.179	6.863	0.009	1.599	1.126–2.273

### Predictive value of circulating ANRIL in identifying DKD

**Fig. 3:**
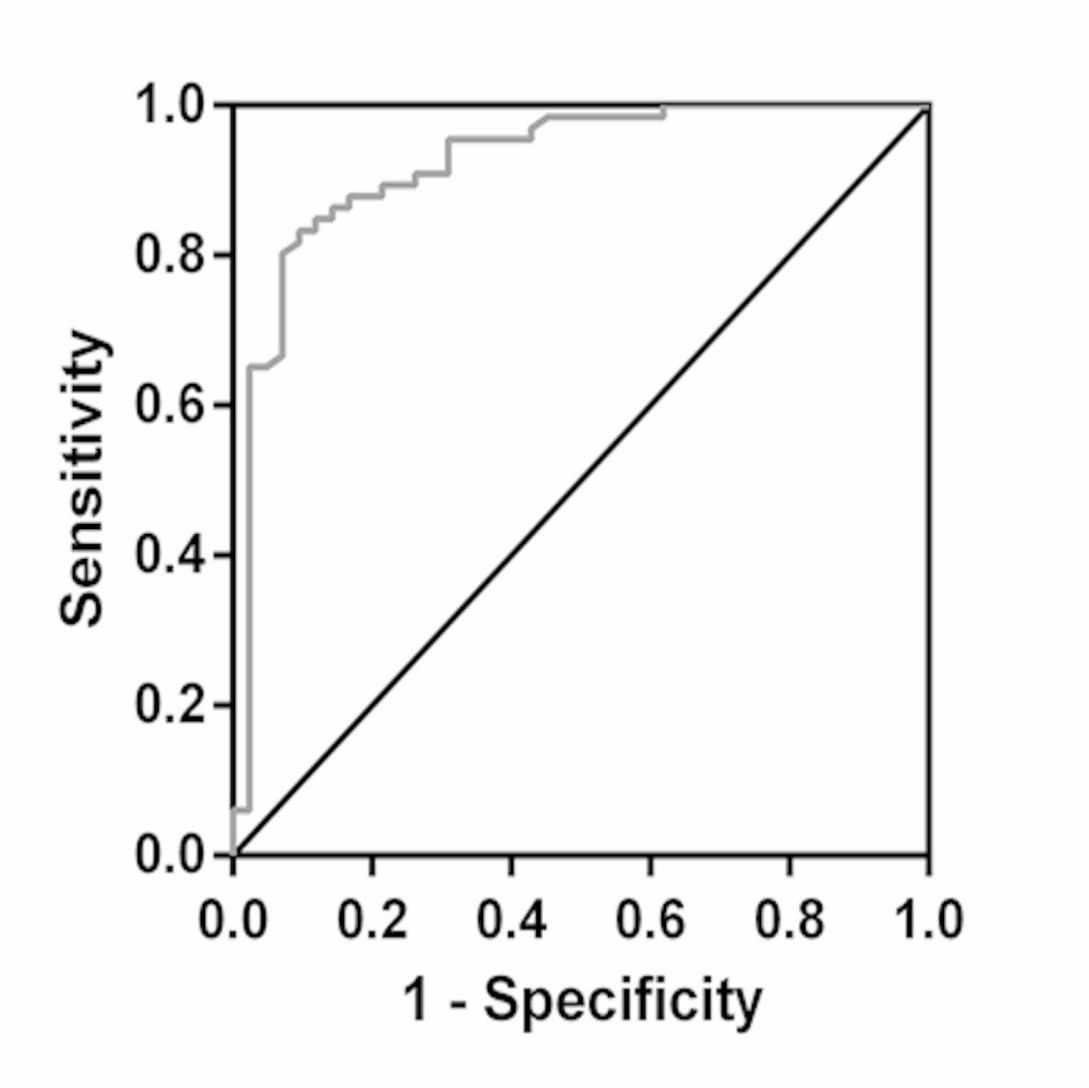
ROC curve analysis of ANRIL in identifying DKD

To confirm the predictive value of circulating ANRIL as the biomarker for the early diagnosis of DKD, the diagnostic value of ANRIL was evaluated by ROC curve analysis. As depicted in Fig. 3, the AUC of ANRIL was 0.922. The sensitivity of ANRIL for predicting DKD was 83.3%. The specificity was estimated at 90.5%. The diagnostic cutoff point was 3.059.

## Discussion

DKD is a common DM complication characterized by a progressive damage of kidney structure and deterioration of renal function, which has become the primary cause of ESRD worldwide [1–3, 8]. Renal fibrosis, a common pathological feature of DKD, characterized by abnormal activation of renal myofibroblasts and excessive accumulation of renal ECM, is majorly caused by EMT/EndMT [5]. DKD remains a main challenging clinical problem in spite of continual progress in treatment and management. Increasing evidences demonstrated that lncRNA plays critical role in the pathogenesis of DKD [14]. LncRNAs are involved in the progression of kidney disease through regulation of many important factors, such as pathologic processes in mesangial cells/podocytes, reactive oxidative species, EMT/EndMT via regulation of diverse targets or functioning as sponges for regulatory microRNAs [14]. Studies have indicated that several lncRNAs induce EMT and renal fibrosis in kidney tissue of DKD animal model and high glucose-stimulated HK2 cells/podocyte via binding to its targeting miRNAs [16, 27, 28]. Furthermore, it has been shown that lncRNA H19 promotes EndMT in TGF-β2-induced fibrosis in human dermal microvascular endothelial cells and in the kidney of streptozotocin-induced diabetic CD-1 mice via regulation of its target miR-29a [17]. Recently there has been more attention about lncRNA ANRIL and its impact on the development of DKD. ANRIL contains 19 exons, spans a region of 126 kb in the antisense orientation of the p15/CDKN2B-p16/CDKN2Ap14/ARF gene cluster [29]. Studies have showed that ANRIL mediates EMT by regulating downstream gene/protein in cancers, such as pancreatic cancer, laryngeal squamous cell carcinoma and renal cell carcinoma [30–32]. ANRIL has also been found to regulate functional and structural alterations in the kidneys in diabetes through controlling the expressions of ECM proteins and VEGF [20]. Another study has suggested that ANRIL silencing alleviates high glucose-induced inflammation, oxidative stress and apoptosis via upregulation of MME in podocytes [26]. In addition, it has been shown that ANRIL promotes pyroptosis and kidney injury in DKD acting as miR-497 sponge [33].

ANRIL in peripheral whole blood, mainly expression in leukocytes, is relatively stable. This present study examines the expression of ANRIL in peripheral whole blood and shows that the expression of ANRIL in peripheral whole blood was significantly upregulated in DKD patients than those in healthy controls and T2DM patients. ANRIL expression showed a positive correlation with Scr, BUN, CysC, urine β2-MG and urine α1-MG, while negatively correlated with eGFR in DKD patients. These above clinical parameters were used to evaluate kidney function status of DKD. Binary regression analysis showed that ANRIL was the risk factor of DKD. The results indicated that ANRIL might be involved in the kidney impairment of DKD; might be play a key role in the pathogenesis of DKD and might be an efficient target for DKD prevention and treatment.

In the early stage, DKD begin from glomerular hyperfiltration, without any clinical symptoms, followed by the development of microalbuminuria. Along with gradual progression, DKD manifests as a clinical syndrome including persistent albuminuria, increased blood pressure, sustained reduction in GFR and increased cardiovascular events [4, 9, 34]. Albuminuria is one of the most characteristic clinical signs in DKD, and is used as an important index for laboratory diagnosis of early DKD and evaluating active and deteriorating condition in DKD [4, 8]. In our study, ANRIL was over-expressed in macroalbuminuria and renal dysfunction groups in comparison with normalbuminuria group. Furthermore, the sensitivity and specificity of ANRIL for predicting DKD were 83.3% and 90.5%, respectively. These results implied that ANRIL can be used as an early diagnostic biomarker for the occurrence of DKD and a predictor for the progression and outcome of DKD in patients. Nonetheless, the influential factors of DKD are diverse. The specific pathogenesis, diagnosis and outcome assessment should be further elucidated.

This is the first study that revealed an independent relationship between ANRIL expression in peripheral whole blood and DKD patients. While, there is still a limitation in this study. Sample size in this study was small, which might result in bias of the results evaluating the association of ANRIL expression and DKD.

In conclusion, our findings provided new evidence that the presence and progression of DKD is associated with an over-expressed ANRIL in peripheral whole blood. Circulating ANRIL is an independent risk factor of DKD and has a highly predictive value in identifying DKD.
